# Self-Assembly in an Experimentally Realistic Model of Lobed Patchy Colloids

**DOI:** 10.1021/acsabm.2c00910

**Published:** 2023-01-25

**Authors:** Remya Ann Mathews Kalapurakal, Brunno C. Rocha, Harish Vashisth

**Affiliations:** Department of Chemical Engineering, University of New Hamphire, Durham, New Hampshire03824, United States

**Keywords:** Self-assembly, polydispersity, lobed colloids, Langevin dynamics, porosity

## Abstract

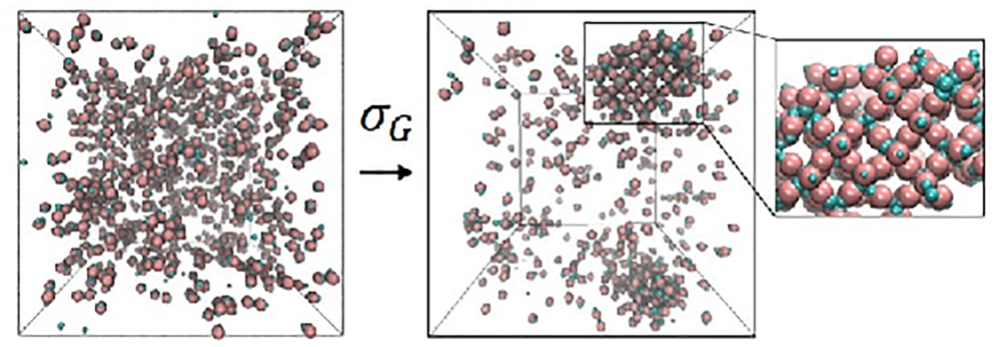

Colloids with lobed architectures have been shown to self-assemble into promising porous structures with potential biomedical applications. The synthesis of these colloids via experiments can be tuned to vary the number and the position of the lobes. However, the polydispersity involving the numbers, sizes, and the dispositions of lobes, that is often observed in particle designs, can significantly affect their self-assembled structures. In this work, we go beyond the uniform lobe size conditions commonly considered in molecular simulations, and probe the effect of polydispersity due to non-uniform lobe sizes by studying self-assembly in three experimentally observable designs of lobed particles (dumbbell, two lobes; trigonal planar, three lobes; and tetrahedral, four lobes), using coarse-grained Langevin dynamics simulations in the NVT ensemble. With increasing polydispersity, we observed the formation of a crystalline structure from a disordered state for the dumbbell system, and a loss of order in the crystalline structures for the trigonal planar system. The tetrahedral system retained a crystalline structure with only a minor loss in compactness. We observed that the effect of polydispersity on the self-assembled morphology of a given system can be minimized by increasing the number of lobes. The polydispersity in the lobe size may also be useful in tuning self-assemblies toward desired structures.

## 1 Introduction

Bottom-up self-assembly of colloidal particles into complex structures has been previously studied under various design conditions including the size, shape, pair-interactions, and surface modifications.^1−7^ The fundamental interactions between these colloidal particles can also be tuned in their assembly.^8,9^ Moreover, the shapes and the surface modifications of uniform colloids provide an additional degree of freedom for tuning the self-assembled structures.^10−16^ These structures find numerous applications as biomaterials,^17,18^ photonic crystals,^19−21^ biosensors,^22^ and smart materials.^23,24^

An important class of self-assembled structures made up of colloidal particles include porous microgels, which are highly desired as tissue engineering scaffolds or substrates.^17,18,25^ The porosity of a microgel could be potentially enhanced by using particles with lobes that provide for additional excluded volume. Such lobed colloidal particles have been synthesized using various techniques including emulsion polymerization,^26−30^ swelling,^31−34^ and other complex processes.^35−42^ We have previously reported that the lobed patchy colloids with multiple lobes lead to porous self-assembled structures primarily due to their non-spherical shape.^43−48^

While the synthesis of lobed colloids suggests polydispersity in several aspects (e.g., lobe size and position),^30^ simulation studies are commonly conducted with monodispersed colloidal particles having uniform lobes. Since this polydispersity affects the self-assembled structures, it is useful to incorporate this metric in models of the lobed particles used in simulations. In this work, we introduce a new model of lobed particles that incorporates the polydispersity in lobe size, as seen in experimentally designed lobed particles.^30^ Our model allows for a comparison with uniformly lobed self-assemblies reported in our previous work,^43−48^ and facilitates an understanding of the effect of the degree of polydispersity on self-assembly in lobed colloids.

## 2 Models and Methods

### 2.1 Model

**Figure 1:**
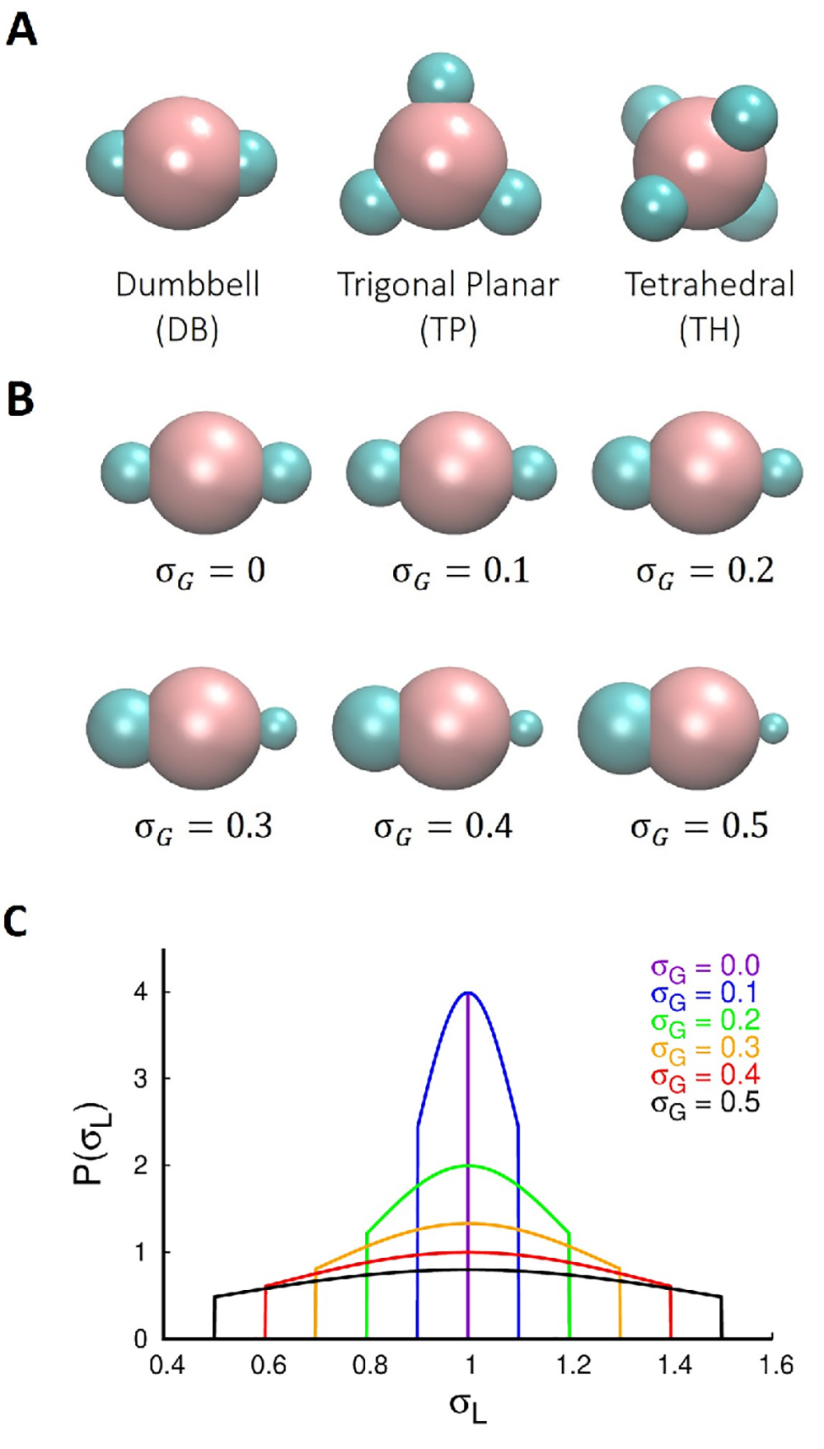
(A) A schematic showing three different types (dumbbell, trigonal planar and tetrahedral) of lobed patchy colloids investigated in this work. (B) A schematic of the dumbbell particle showing the minimum (the right-side lobe) and the maximum (the left-side lobe) lobe size spanning six different values of σ_*G*_. (C) The Gaussian distribution used in sampling the lobe size is shown. The distribution is truncated at the minimum and the maximum allowed values.

The lobed colloidal particles with uniform lobes were modeled as a combination of two entities - a seed (diameter, σ_*S*_; pink sphere in Figure 1A), and a lobe (diameter, σ_*L*_; cyan sphere in Figure 1A).^43−48^ In our previous studies,^43,46,47^ we fixed the diameter of the lobe and the seed as σ_*L*_ = 1 and σ_*S*_ = 2σ_*L*_ (Figure 1A). To incorporate polydispersity in the lobe size, we randomly chose the size of each lobe from a Gaussian distribution having a mean (μ_*G*_) and a standard deviation (σ_*G*_). We sampled the lobe size from the interval [μ_*G*_ – σ_*G*_, μ_*G*_ + σ_*G*_], which accounts for the smallest and the largest lobe size possible for different values of σ_*G*_ (Figure 1B and C). We fixed μ_*G*_ = σ_*L*_ = 1 (the diameter of the lobe for the monodisperse case) and studied the effect of varying σ_*G*_ on the self-assembled structures. The values of σ_*G*_ chosen for the study were 0.1, 0.2, 0.3, 0.4, and 0.5. A σ_*G*_ value greater than 0.5 was not considered in this study, since a lobe size less than 0.5 would not be stable in experiments.^30^

### 2.2 Interparticle Potential

The non-bonded interactions between a pair of lobed particles were taken to be the sum of seed–seed (S–S), seed–lobe (S–L), and lobe–lobe (L–L) interactions. The repulsive seed–seed and seed–lobe interactions were modeled via a surface shifted Lennard-Jones potential, as given by eq 1:
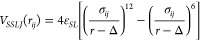
1where ϵ_*SL*_ is the depth at the energy minimum for a pair of entities (either seed–seed or seed–lobe) *i* and *j*, having diameters σ_*i*_ and σ_*j*_, and separated by a distance of *r*_*ij*_.  is the closest non-penetrable distance between these two particles, and Δ = σ_*ij*_ – 1 shifts the potential to the surface of a given particle. The interactions are effective when *r*_*ij*_ is within a cutoff range of (*r*_*cut*_ + Δ), beyond which the particles do not interact. The value of *r*_*cut*_ is set to 2^1/6^σ_*ij*_ and ϵ_*SL*_ is set to 1 *k*_*B*_*T* for all pairs of entities interacting via the SSLJ potential.

The attractive lobe–lobe interactions were modeled based on the Lennard-Jones potential (eq 2).
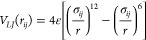
2

The ϵ value is set to 3 *k*_*B*_*T* for a pair of lobes interacting within a cutoff distance (*r*_*cut*_ = 3σ_*ij*_) to be consistent with our previous studies.^43−48^ The unit of length (σ) is taken as 1, which is the mean size of a given lobe (σ_*L*_). We probed the self-assembly behavior of three different systems of lobed particles (dumbbell, DB; trigonal planar, TP; and tetrahedral, TH) (Figure 1A).

### 2.3 Simulation Setup

We simulated each system of lobed particles by using coarse-grained Langevin dynamics simulations (in the NVT ensemble) and using the HOOMD-blue software.^49^ Each system was made up of 1000 particles, each differing in the sizes of its lobes, which were sampled randomly^50^ from a Gaussian distribution (Figure 1C). Therefore, each particle is distinct from other particles in a given polydispersed system. As a result, the interaction parameters were uniquely assigned to each pair of interacting entities.

We studied each system at five different values of the reduced temperature (T* = 0.2, 0.4, 0.6, 0.8, and 1.0). We performed all simulations in cubic simulation domains with periodic boundary conditions applied in all directions. The volume fraction of each system was fixed at ϕ = 0.1
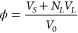
3where the constants *V*_*S*_ and *V*_*L*_ are the volumes of the seed and the lobe in each particle, *N*_*L*_ is the number of lobes present on each lobed particle, and *V*_0_ is the volume of the simulation domain. To maintain consistent sizes in simulation domains of all systems, we considered a constant value of V_*L*_, equal to the volume of a lobe with a diameter σ_*L*_ = 1. We note that the volume fraction calculated using the actual diameter of the polydisperse lobes was confirmed to be ϕ ≈ 0.1 in each case. The corresponding dimensions of the simulation domain for each case are 75 σ_*L*_ (DB), 78 σ_*L*_ (TP), and 80 σ_*L*_ (TH). Each system was equilibrated for 5 × 10^7^ steps, followed by another 5 × 10^7^ production steps, during which the equilibration properties of each system were monitored. A time-step of 0.005 was used in all simulations.

### 2.4 Analyses Techniques

We carried out the visual characterization of each system using the Visual Molecular Dynamics (VMD) software.^51^ The metrics used in quantitative analyses are briefly explained below.

#### 2.4.1 Radial Distribution Function (RDF)

We determined the spatial arrangement and phase characteristics of each system by calculating the radial distribution function using eq 4
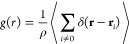
4The RDF gives the local density and the arrangement of particles with respect to the distance from a reference particle (**r**) in comparison to the bulk density (ρ).

#### 2.4.2 Relative Neighbor Orientation (θ_*jik*_)

We also calculated the relative position of two nearest neighbors of a particle. For a particle *i*, having two nearest neighbors, *j* and *k*, the relative neighbor orientation is given by the angle formed between the vectors  and , where the vectors  and  give the distance between the corresponding pairs of particles (Figure S1). Further, we computed the probability of the relative neighbor orientation, P(θ_*jik*_). In addition to RDF, this explains the spatial arrangement of the particles with respect to their orientations around a reference particle, the particle *i*.

#### Average Number of Bonds Per Lobe 

2.4.3

We calculated the average number of bonds formed by each lobe of a given particle. We assumed that the lobes in a pair of particles formed an interparticle bond via the LJ potential when the lobes are within the cutoff distance (*r*_*cut*_ = 3σ_*ij*_). In addition to this, we also calculated the distribution of the number of lobes with a particular lobe size forming a given number of bonds. These are referred to as the lobe-size and the bond distributions.

#### 2.4.4 Porosity Analysis

We used the pore size distribution (PSD) as the metric to evaluate the porosity of the self-assembled structures. We computed PSD by extracting the largest possible cuboids from the self-assembled structures and then using the Zeo++ software^52,53^ to perform this analysis. The probe radius used in the PSD calculations was equal to 1/2 μ_*G*_, consistent with our previous studies.^43−45,47^

## 3 Results and Discussion

### 3.1 State Diagram of Self-Assembled Morphologies

**Figure 2:**
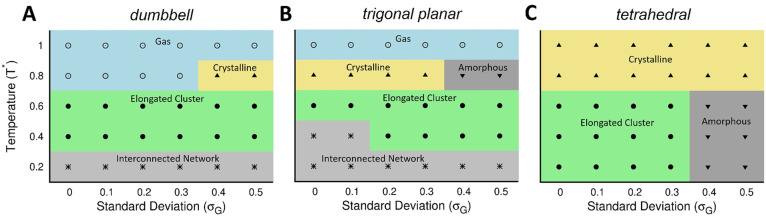
State diagrams showing the self-assembled phases exhibited by the (A) dumbbell, (B) trigonal planar, and (C) tetrahedral lobed particles at various conditions of reduced temperature (T*) and polydispersity (σ_*G*_).

We categorized each system based on the final self-assembled morphology (Figure 2). Specifically, we observed interconnected networks, elongated clusters with different microstructures, crystalline and amophous aggregates, and a disordered gas phase. The interconnected networks made up of the DB particles contained 5, 6, and 7 membered rings (Section 3.2.1), as also reported in our previous study.^43^ These rings were different from the chains observed in our other previous studies,^46,48^ where the particles were functionalized or charged. In both cases, the chains were longer. The small size of the functionalized lobe restricted the bending and branching in the chains formed by the functionalized particles,^46^ while the electrostatic repulsion reduced the bending of the chains in the charged system.^48^ The TP particles formed interconnected networks with a hexagonal arrangement of the particles, and significant branching (Section 3.2.2). The elongated clusters formed by the DB particles consisted of a higher degree of trigonal prismatic ring-like arrangements, as also reported in our previous study,^43^ while the TP and TH particles displayed hexagonal arragements. At higher temperatures, we observed compact crystalline or amorphous structures for all three types of particles. The specific conditions for which these structures were observed, and the changes observed due to polydispersity are discussed next.

### 3.2 Effect of Polydispersity on Self-Assembled Morphologies

We investigated the effect of polydispersity in lobe size on the self-assembled structures for a range of temperature values. The structures reported in the state diagram (Figure 2) were quantitatively analyzed. The metrics used in these analyses included the radial distribution function (RDF), the relative neighbor orientation (θ_*jik*_), the average number of bonds formed per lobe (), and the lobe-size and bond distributions.

#### 3.2.1 Self-Assembled Morphologies: Dumbbell Particles

**Figure 3:**
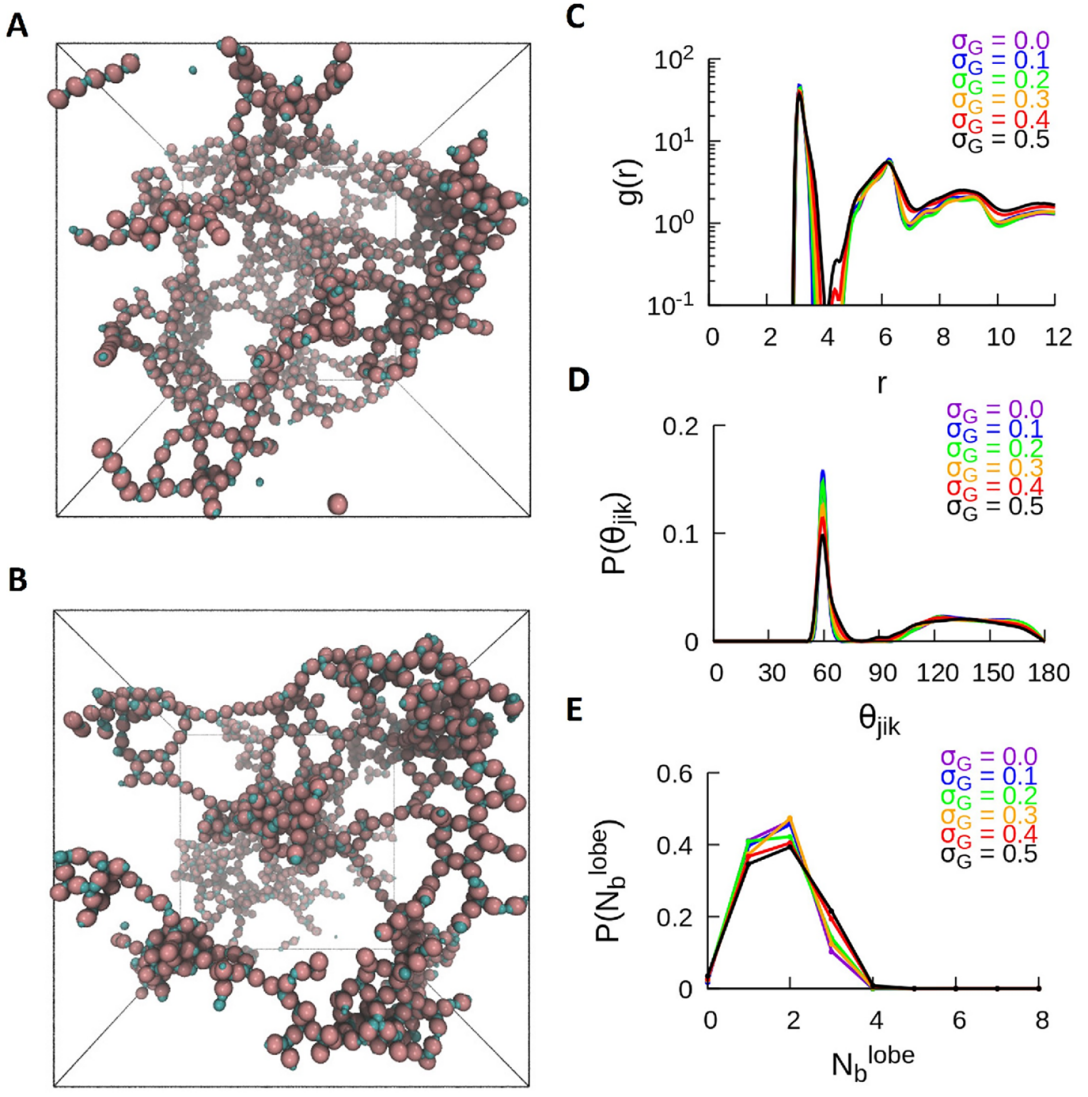
Self-assembly of the DB particles at T* = 0.2. Shown are snapshots of the interconnected networks at (A) σ_*G*_ = 0.1 and (B) σ_*G*_ = 0.5. The traces from the quantitative analyses for the DB system at T* = 0.2 and for all values of σ_*G*_ are also shown: (C) RDF, (D) P(θ_*jik*_), and (E) P().

At the lowest reduced temperature (T* = 0.2), the DB particles self-assembled into interconnected networks consisting of branched chains and rings (Figure 3A and B). We observed that the polydispersity did not significantly affect the internal morphology of the system. This was quantitatively confirmed by the RDF and P(θ_*jik*_) calculations (Figure 3C and D), where we observed no differences in the traces for different values of σ_*G*_, except for a marginal decrease in the nearest neighbors in the first coordination shell. At lower polydispersity values, we also observed that on an average one to two bonds were formed per lobe (Figure 3E). The single bond per lobe corresponds to the ring motifs and occasionally to the chains found in the morphology, while the two bonds were responsible for branching in the network.

With an increase in polydispersity, the number of bonds formed per lobe varied to a larger extent. At σ_*G*_ > 0.3, the smaller lobes (σ_*L*_ < 1) formed lesser number of bonds (0 or 1), while the larger lobes (σ_*L*_ > 1) formed a higher number of bonds (3 or 4) (Figure S2 A-C).

At T* = 0.4 and 0.6, the elongated clusters largely consisting of rings were observed. The data on the spatial and the orientational arrangements of these structures are comparable for σ_*G*_ ≤ 0.3 (Figures S3 and S4). The trigonal prismatic arrangement contributed to the branching occurring between these rings, thereby leading to an increased probability of the formation of three bonds per lobe. At σ_*G*_ > 0.3, the system gradually evolved into a crystalline ordered arrangement, as indicated by a second peak in RDF at *r* < 5 and a second peak at ∼90° for P(θ_*jik*_) (red and black traces for σ_*G*_ = 0.4 and 0.5, respectively; Figure S4). Owing to the presence of the larger lobes, the P trace showed an increase in probability for the formation of four bonds per lobe. However, a higher number of particles also formed zero bonds due to the smaller lobes.

**Figure 4:**
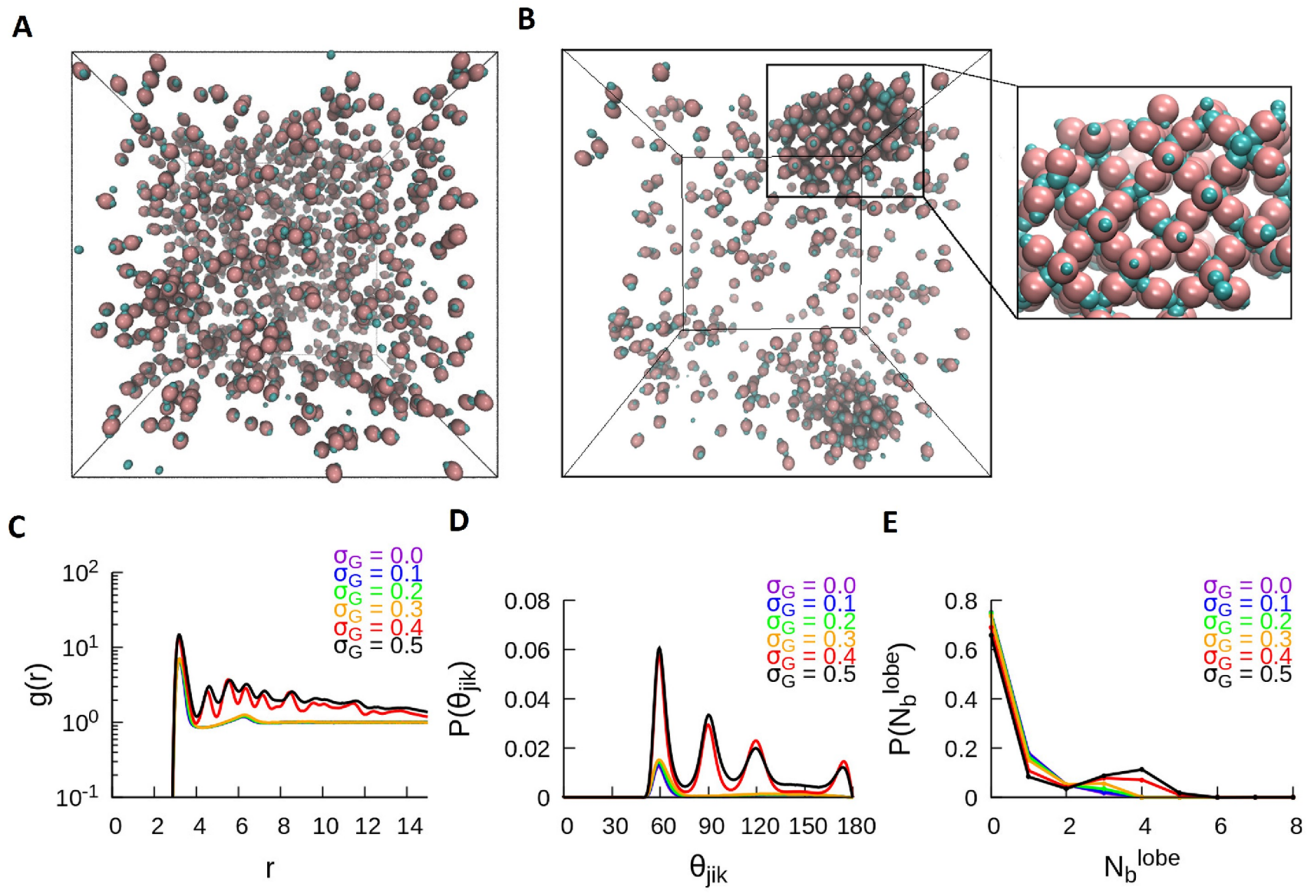
Self-assembly of the DB particles at T* = 0.8. (A) Shown is the snapshot of a disordered gaseous state for σ_*G*_ = 0.1 and (B) the snapshot of a crystalline structure for σ_*G*_ = 0.5, where the inset shows the top view of the crystal structure. For T* = 0.8, the traces of various metrics similar to Figure 3 for all values of σ_*G*_ are shown (C) RDF, (D) P(θ_*jik*_), and (E) P().

At a higher temperature (T* = 0.8) and for σ_*G*_ < 0.3, the system became disordered into a gaseous state, while for σ_*G*_ = 0.4 and 0.5, the system led to a crystalline structure (Figure 2A). We have previously shown that the DB particles self-assembled into crystalline structures for varying seed to lobe ratios.^44^ Similar to that work, with an increase in polydispersity, the seed-lobe ratio becomes favorable with the increasing lobe size, thereby facilitating the formation of the crystalline structures. The nucleation of the crystal structure from a disordered system is shown in Figure 4B. The inset shows the top view of a typical crystalline arrangement. The well-ordered peaks in the RDF and P(θ_*jik*_) traces (Figures 4C and 4D) confirmed the crystalline morphology of the system. The flat curves observed for lower σ_*G*_ (violet to orange traces for σ_*G*_ = 0.1 to 0.3, respectively) indicated the gaseous phases. At a higher polydispersity, the lobes smaller or larger than the mean lobe size (σ_*L*_ = 1) are equally probable. The presence of the smaller lobes may cause defects in the crystalline structure. At T* = 1.0, the system existed as a gas at all conditions of polydispersity (Figure S5). We have also carried out additional independent simulations of the system at the same thermodynamic conditions and observed consistency among the structures obtained (Figure S6), as quantified by the metrics computed (Figure S7).

#### 3.2.2 Self-Assembled Morphologies: Trigonal Planar Particles

At T* = 0.2, we observed the formation of interconnected networks (Figures 2B and S8). However, the arrangement of the particles was different from that of the DB system. Specifically, the particles arranged into a two-dimensional triangular arrangement with elongation and branching that extended to a network-like arrangement. Similar to the DB system, the increase in polydispersity did not significantly alter the morphology of the system. However, a difference in the number of bonds formed per lobe was observed (Figure S9). For the lower polydispersity values, the majority of the particles formed one to two bonds per lobe, along with three bonds per lobe, leading to branching in self-assemblies. However, at the higher polydispersity values, the particles formed no bonds (smaller lobes) or three bonds (larger lobes) per lobe. To a small extent, this led to a change in the triangular arrangement of the particles into branching.

**Figure 5:**
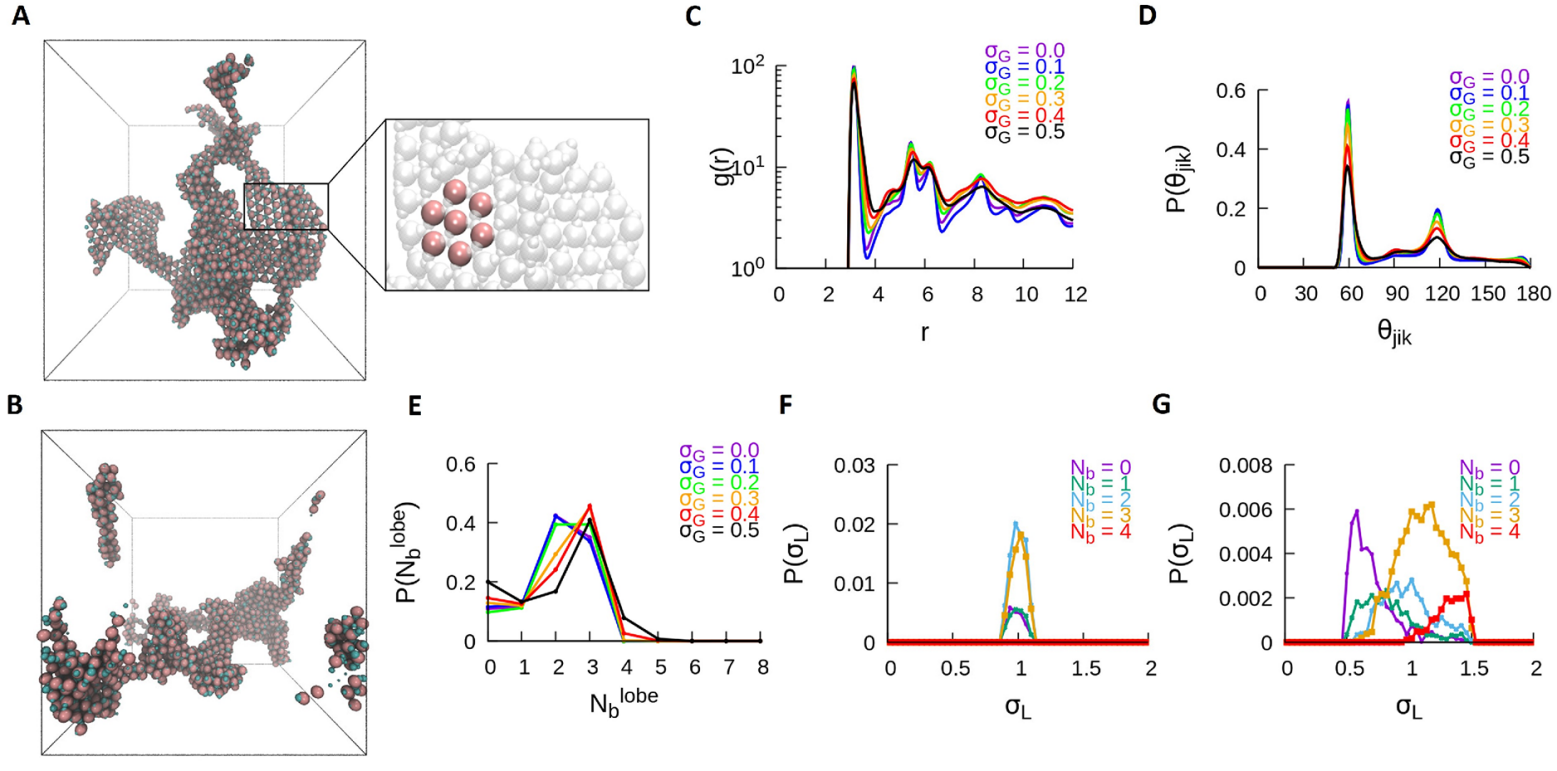
Self-assembly of the TP particles at T* = 0.4. (A) Shown is a snapshot of the elongated clusters with a local structure of two-dimensional sheets for σ_*G*_ = 0.1. The inset shows the microstructure of a typical two-dimensional sheet. The particles forming the hexagonal arrangement around a locus particle are depicted. (B) A snapshot of the elongated clusters for σ_*G*_ = 0.5. Shown are the traces of the quantitative metrics computed for the TP system at T* = 0.4, for all values of σ_*G*_: (C) RDF, (D) P(θ_*jik*_), (E) P(), and the lobe-size and bond distributions for (F) σ_*G*_ = 0.1 and (G) σ_*G*_ = 0.5.

At T* = 0.4, the elongated clusters were observed (Figure 5A and B), where the microstructure is similar to the networks in which the particles formed small two-dimensional triangular sheets with branching. A representative snapshot of the system is shown in Figure 5A, where the inset shows a hexagonal arragement of the particles. The RDF and P(θ_*jik*_) traces (Figure 5C and D) show well-ordered peaks confirming a hexagonal arrangement of the particles. At the lower values of polydispersity, two-dimensional sheets were predominant, resulting on average in two bonds per lobe. Three bonds per lobe were also observed, which is indicative of a higher level of branching (Figure 5E and F). However, with increasing polydispersity, we observed a decrease in the probability of two bonds per lobe, with an equivalent increase in zero, three, and four bonds per lobe, similar to the previous cases (Figure 5G). This resulted in three-dimensional clusters in comparison to the two-dimensional sheet.

With an increase in temperature (T* = 0.6 and 0.8), the system exhibited a crystalline arrangement. At both temperatures, we observed a loss of the crystalline order with increasing polydispersity, which is marked by a decrease in the peak height in the RDF and P(θ_*jik*_) traces as well as a change in P, similar to the previous cases (Figures S11 and S12). However, at T* = 0.8, the mobility in the particles was higher. Along with the increase in the number of smaller lobes, this led to a larger number of particles that did not self-assemble. Therefore, the cluster at these conditions (T* = 0.8 and σ_*G*_ = 0.5) coexisted with a gaseous phase. We observed that P showed an increase at zero and a decrease for the other values on approaching σ_*G*_ = 0.5. A slightly increasing peak for four bonds per lobe (at σ_*G*_ = 0.5) was due to the presence of the larger lobes. At T* = 1.0, the system existed in a gas phase at all conditions of polydispersity (Figure S13).

#### 3.2.3 Self-Assembled Morphologies: Tetrahedral Particles

With the increase in the number of lobes, the formation of well-ordered self-assemblies was favorable. In this system, we observed the formation of the elongated clusters (Figure 2C) up to T* = 0.6, with a relatively higher degree of spatial order in comparison to the other two types of systems made up of the DB and TP particles (Figure S14). Between T* = 0.2 and 0.6, we found an increase in the crystalline order in self-assembled morphologies, primarily due to the dynamics and rearrangements allowed under higher temperatures. At the lower values of polydispersity, the most favorable number of bonds formed per lobe was two, leading to the elongated clusters. With an increase in polydispersity, the larger lobes formed three bonds per lobe with an equivalent increase in smaller lobes forming no bonds. This shift in P with polydispersity manifested as the loss in spatial order, as evidenced by the RDF and P(θ_*jik*_) traces at various conditions (Figures S15–S17).

The crystalline structures formed by the TH particles were reported to have a hexagonal arrangement with alternating layers.^43^ This structure was stabilized when the majority of the particles formed three bonds per lobe with the neighboring particles. With temperature approaching T* = 0.6, we observed that the systems at the lower polydispersity values transitioned toward this arrangement. This behavior is prominent at T* = 0.8, where we observed a higher level of ordering even up to σ_*G*_ = 0.4. Even though the peak heights in the RDF and P(θ_*jik*_) traces reduced with polydispersity, the spatial order was not entirely lost. At σ_*G*_ = 0.5, where the percentage of the smaller lobes increased, the number of monomers forming no bonds in the system also increased (Figure S18).

**Figure 6:**
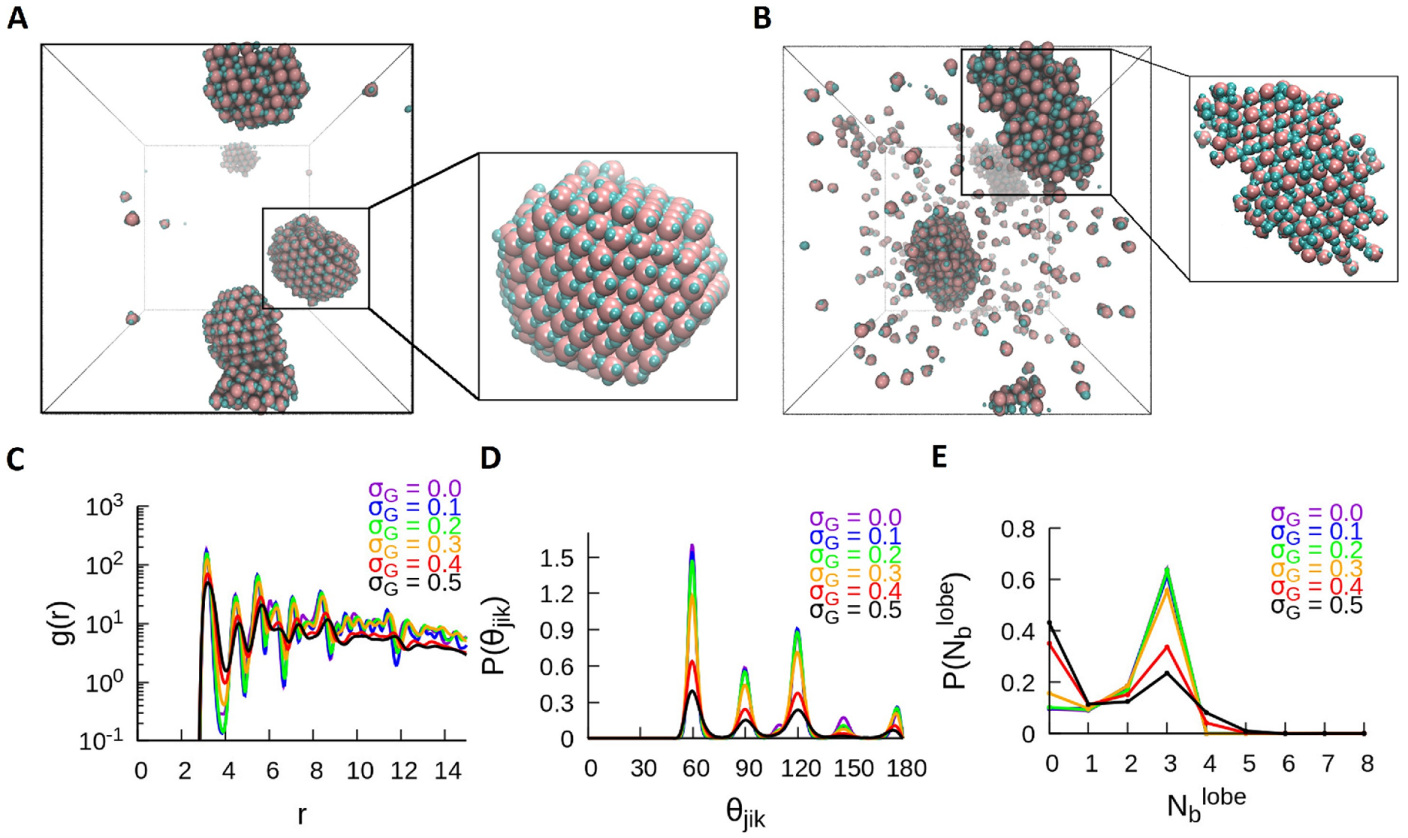
Self-assembly of the TH particles into crystalline structures at T* = 1.0. (A) Shown is a snapshot of the compact three-dimensional crystal for σ_*G*_ = 0.1, where the inset shows the ordered structure. (B) Shown is a less compact crystalline structure for σ_*G*_ = 0.5, where the inset shows the top view of the crystal. Shown are the traces of the quantitative metrics computed for the TH system at T* = 1.0 for all values of σ_*G*_: (C) RDF, (D) P(θ_*jik*_), and (E) P().

At T* = 1.0, where a disordered state was observed for the DB and the TP particles, the TH system stabilized a well-ordered crystalline structure at the lower values of polydispersity. The inset in Figure 6A shows a typical three-dimensional ordered crystal formed at this condition. With an increase in polydispersity and owing to the higher kinetic energies of the particles, the crystal structure coexisted with monomers, on approaching σ_*G*_ = 0.5, as observed for the DB and TP systems as well. The inset in Figure 6B shows the top view of this crystalline structure. Importantly, the cluster still retained some level of spatial order even at a higher value of polydispersity (Figure 6C and D). Therefore, we observed that with increasing the number of lobes, the effect of polydispersity on the self-assembled structures also reduced. The variation in P caused the reduction in the peak heights for the RDF and P(θ_*jik*_) traces (Figures 6C–E and S19).

#### 3.2.4 Pore Size Distribution of Self-Assembled Structures

The PSD data for the DB particles (Figure S20A) are characterized by broad peaks for all σ_*G*_ conditions, which indicates that the pore sizes observed in these structures are heterogeneous. The estimated pore diameters vary between 3.0 μ_*G*_ and 8.5 μ_*G*_, and their probabilities are well-distributed within this range. The variations in σ_*G*_ were observed to affect the pore size distributions. For instance, the range of pore diameters changes from 4.0 μ_*G*_ – 8.5 μ_*G*_ to 4.0 μ_*G*_ – 6.5 μ_*G*_, when σ_*G*_ changes from 0.1 to 0.2. However, a specific pattern for the influence of σ_*G*_ on the pore size distribution was not observed. The PSD data for the TP particles (Figure S20B) are also characterized by broader peaks for all σ_*G*_ conditions, similar to those for the DB particles. The estimated pore diameters vary between 1.8 μ_*G*_ and 7.0 μ_*G*_, which shows a decrease in the overall porosity when compared to the structures obtained from the DB particles. In contrast, the PSD data for the TH particles (Figure S20C) are characterized by sharper and narrower peaks for all σ_*G*_ conditions, which suggests that the pore sizes observed in these structures are homogeneous. The estimated pore diameters vary between 1.2 μ_*G*_ and 3.5 μ_*G*_, a significant decrease in the overall porosity when compared to the structures obtained from the DB and TP particles. The variations in σ_*G*_ for the TH particles did not cause significant variations in the pore size distributions.

The polydispersity can affect the self-assembly of the lobed particles depending on the conditions (temperature and the number of lobes). Our work explains the structural changes that can be expected in an experimental system depending upon the degree of polydispersity. Our results also suggest how the polydispersity can be tuned to attain a desired structure, for example, the crystalline structure for the DB system. However, we also observed that while variations in the polydispersity of the systems may affect their pore size distributions (Figure S20), a patterned correlation between σ_*G*_ and PSD could not be identified, at those temperature conditions where larger porous structures are formed.

These results are potentially useful in the experimental design of porous hydrogel-like scaffolds for tissue engineering applications. For example, we observed the formation of several structures (interconnected networks, elongated clusters, and amorphous/crystalline structures) in different systems. While ordered crystalline structures are desirable for their fixed pore size and periodicity, they may not be always suitable for designing porous hydrogel-like scaffolds, owing to their small pore diameters. However, by changing the seed to lobe ratio and functionalizing the lobes, the pore diameters of the crystalline structures could be significantly increased, while also retaining the periodicity. On the other hand, interconnected networks and elongated clusters can be used as suitable candidates to build porous hydrogel-like scaffolds. These structures pose a higher possibility to form larger pores (intra-network and inter-network pores) that could enhance cell-diffusion and growth. The structures observed at very low temperatures can be mimicked by quenching the temperature of the system during self-assembly, thereby forming interconnected networks due to the limited diffusion of the particles. Therefore, the studies presented on the lobed particles are useful in understanding the system morphology and interaction, which is crucial in designing suitable structures for tissue engineering applications.

## 4 Conclusions

We have reported results on the self-assembly of lobed patchy colloids using an experimentally realistic model that incorporates the polydispersity in the lobe size^30^ and probed the effect of the polydispersity in self-assembly of these models. In this model, the size of each lobe was randomly chosen from a Gaussian distribution, where the standard deviation was taken as the measure of polydispersity. We observed the formation of various self-assembled structures like interconnected networks and elongated clusters with different local arrangements including crystalline and amorphous clusters, and a disordered gas phase. With an increase in polydispersity and the equivalent change in the lobe size, the average number of bonds formed per lobe decreased for smaller lobes and increased for larger lobes, in comparison with the monodispersed cases. This variation led to an increase or a decrease in the spatial order within the self-assembled structures. An increased polydispersity condition also led to the formation of a crystal structure from a disordered phase for the DB particles. We also observed that crystalline structures formed by the TP particles failed to retain the spatial order with an increase in polydispersity. However, polydispersity did not substantially affect the spatial order of the crystal structures formed by the TH particles, despite the loss in compactness observed in these structures. Therefore, we show that polydispersity can be used to tune the self-assembly of desired structures for tissue engineering applications.

## Supporting Information Available

Additional analyses for each system are included in Figures S1–S20 (PDF)
