# Supplementary material for: Self-Assembly in an Experimentally Realistic Model of Lobed Patchy Colloids
Source: ACS Appl Bio Mater. 2023 Jan 25;7(2):535–42. doi: 10.1021/acsabm.2c00910 (PMC10880053; doi:10.1021/acsabm.2c00910)
Supplement: Supplementary file 1 — mt2c00910_si_001.pdf [file mt2c00910_si_001.pdf]

SUPPORTING INFORMATION

Self-assembly in an Experimentally Realistic Model of Lobed Patchy Colloids

Remya Ann Mathews Kalapurakal, Brunno C. Rocha, and Harish Vashisth*

Department of Chemical Engineering, University of New Hampshire, Durham, NH, USA

E-mail: harish.vashisth@unh.edu

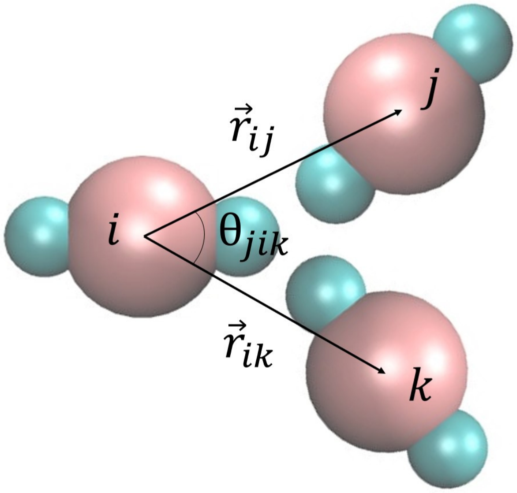

Figure S1: A schematic depicting the angle (θ_{jik}) used in defining the relative neighbor orientation. For a reference particle i, having two nearest neighbors j and k, located at the distances $\vec{r}_{ij}$ and $\vec{r}_{ik}$ respectively, θ_{jik} gives the relative arrangement of the particles j and k with respect to the particle i.

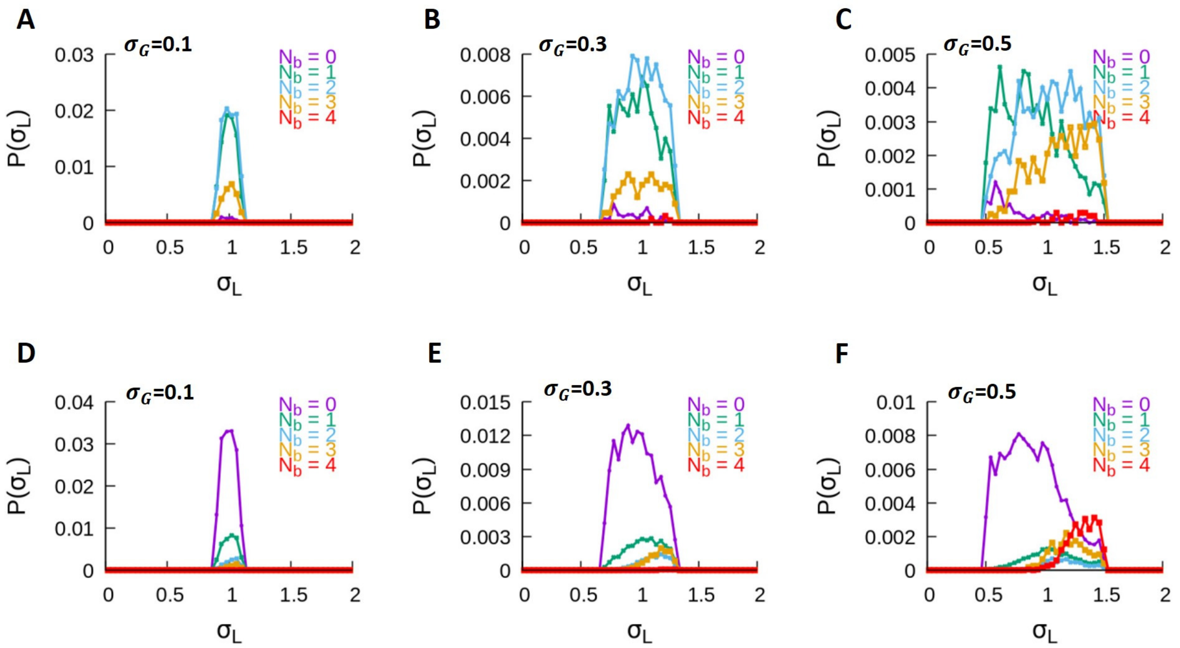

Figure S2: Data for the dumbbell (DB) system at T^* = 0.2 (panels A, B, and C) and T^* = 0.8 (panels D, E, and F). Shown are the distributions of the probability of the lobes of a particular size for forming a specific number of bonds (N_b^{lobe}) at three different values of standard deviation (σ_G = 0.1, 0.3, and 0.5).

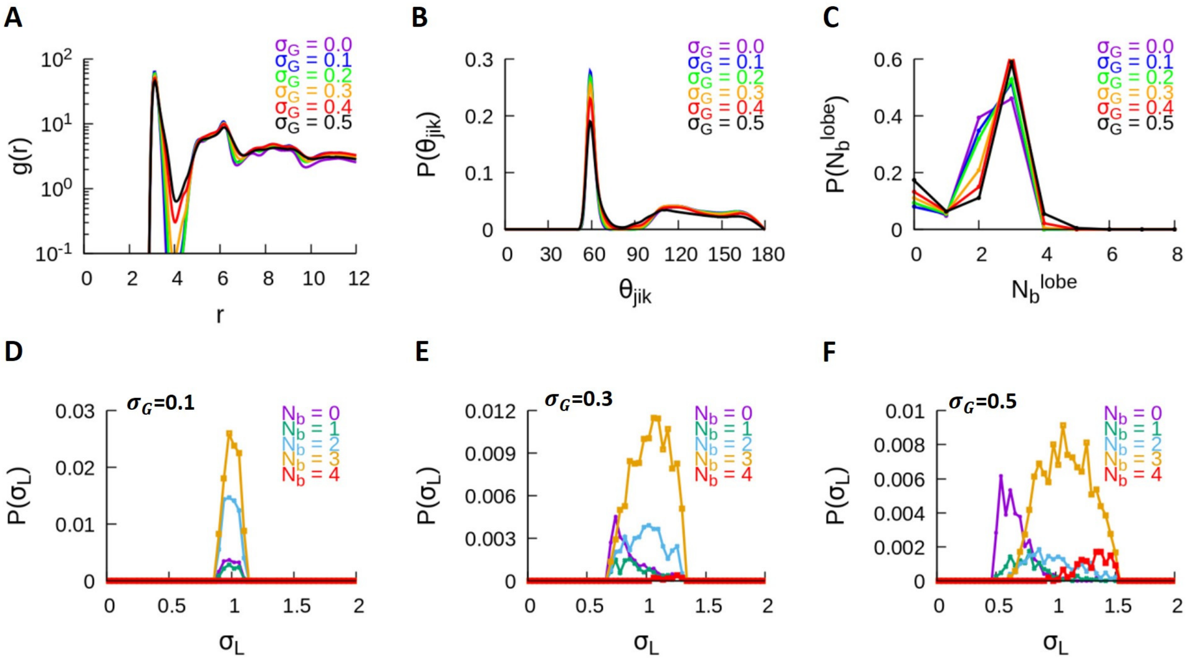

Figure S3: Data for the DB system at $T^{*} = 0.4$. Shown are the traces from the quantitative analyses for all values of σ_G: (A) RDF, (B) P(θ_{jik}), (C) P(N_b^{lobe}), and (D-F) the lobe-size and bond distributions for three different values of standard deviation (σ_G = 0.1, 0.3, and 0.5).

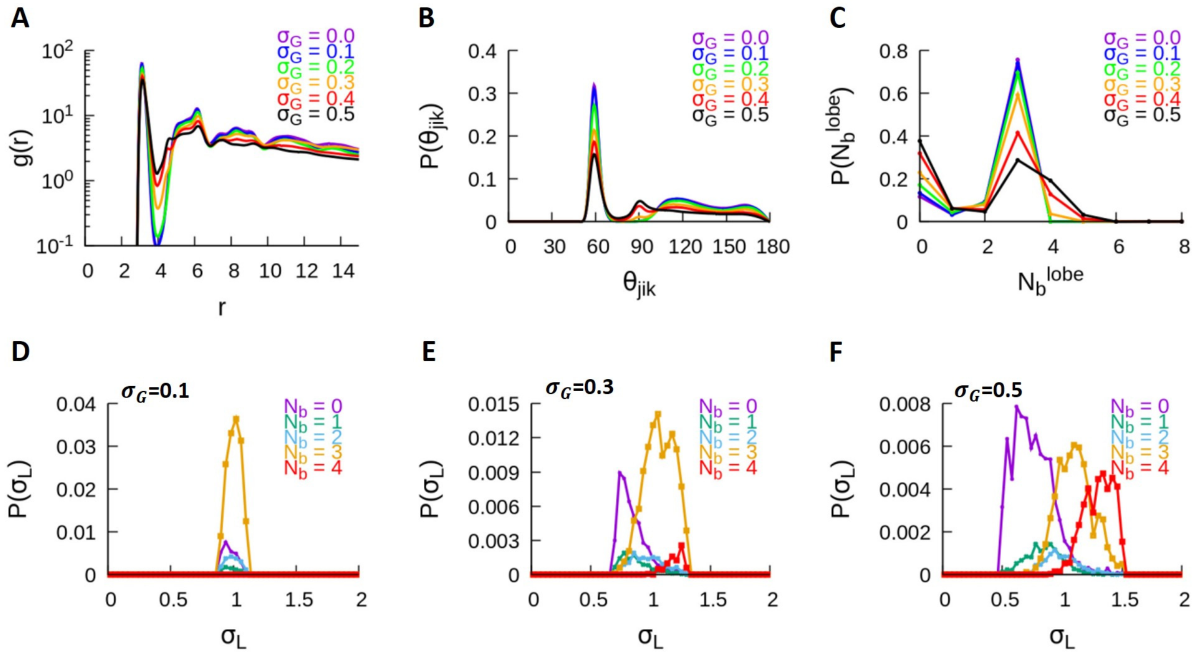

Figure S4: (A-F) Data similar to Figure S3 are shown for the DB system at $T^* = 0.6$.

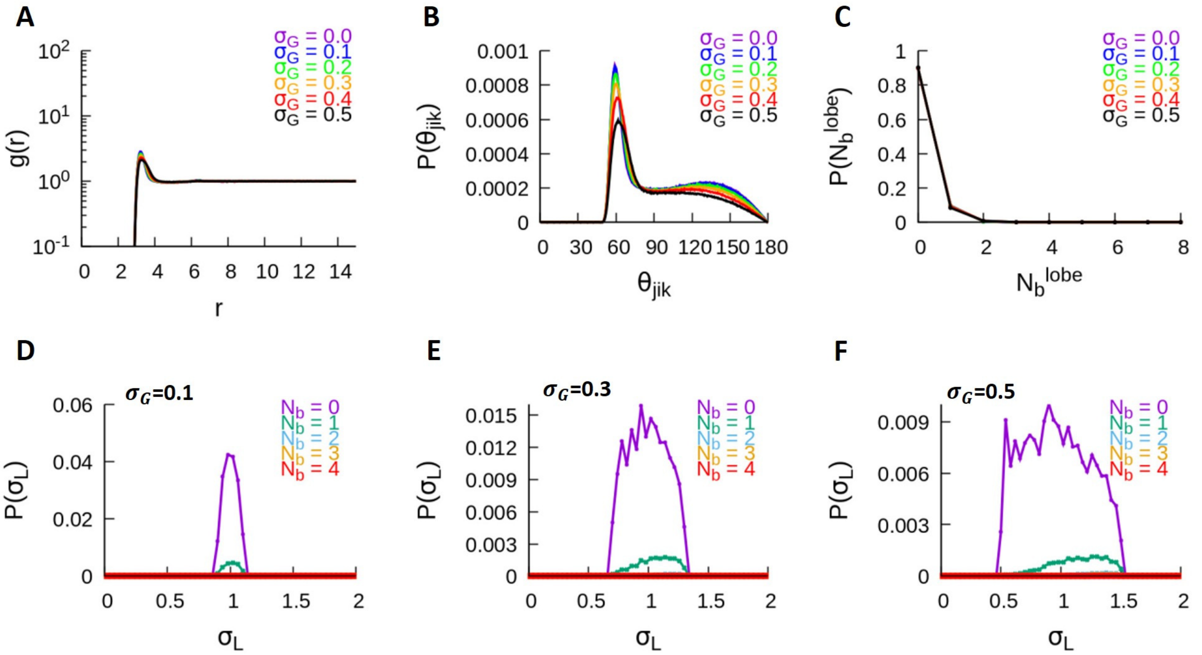

Figure S5: (A-F) Data similar to Figure S3 are shown for the DB system at $T^* = 1.0$.

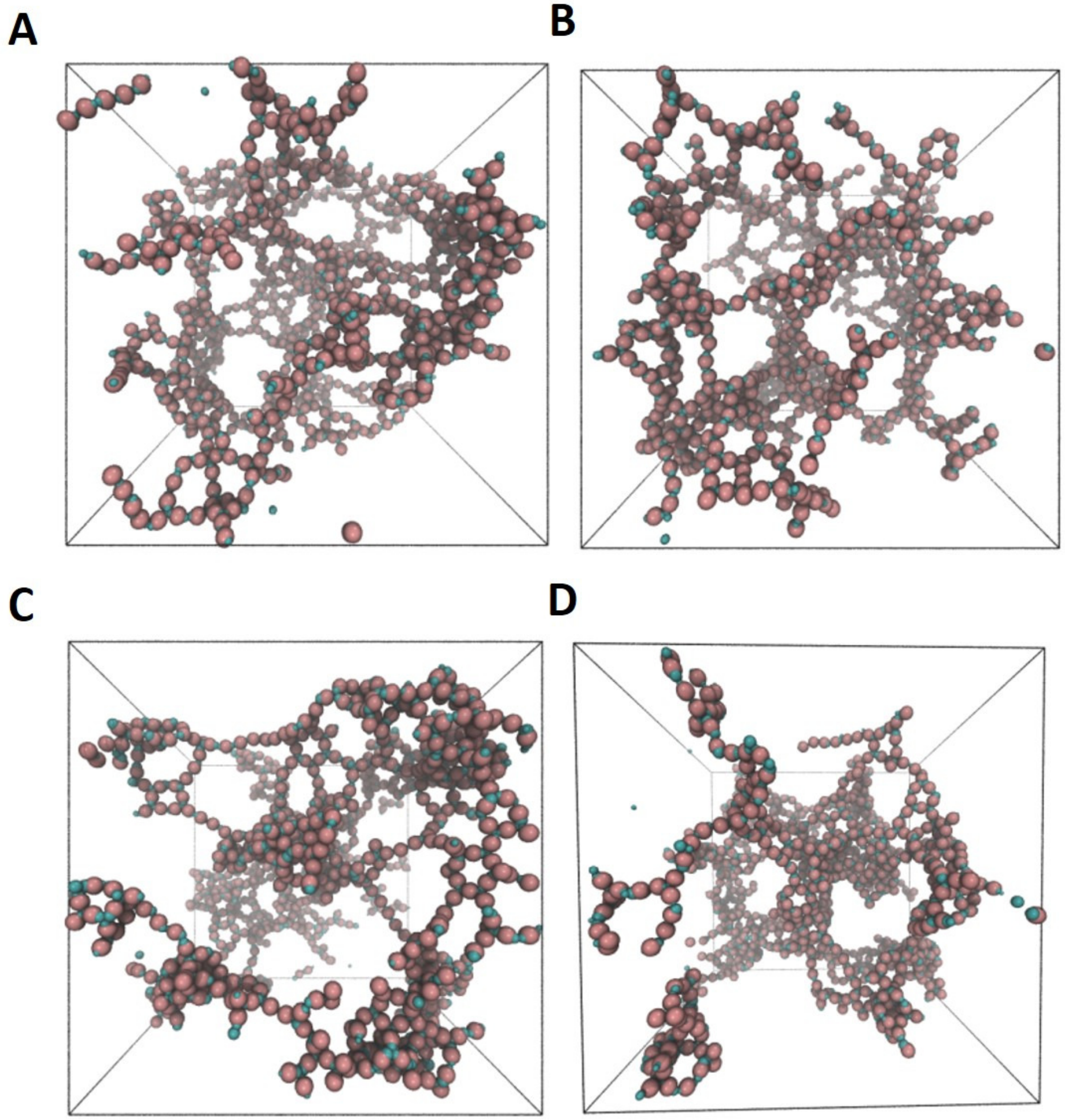

Figure S6: A comparison of results between multiple runs of the DB system at $T^* = 0.2$ and $\phi = 0.1$. Shown are the snapshots of the interconnected networks observed for different conditions of σ_G: (A) $\sigma_G = 0.1$, run1, (B) $\sigma_G = 0.1$, run2, (C) $\sigma_G = 0.5$, run1, (D) $\sigma_G = 0.5$, run2.

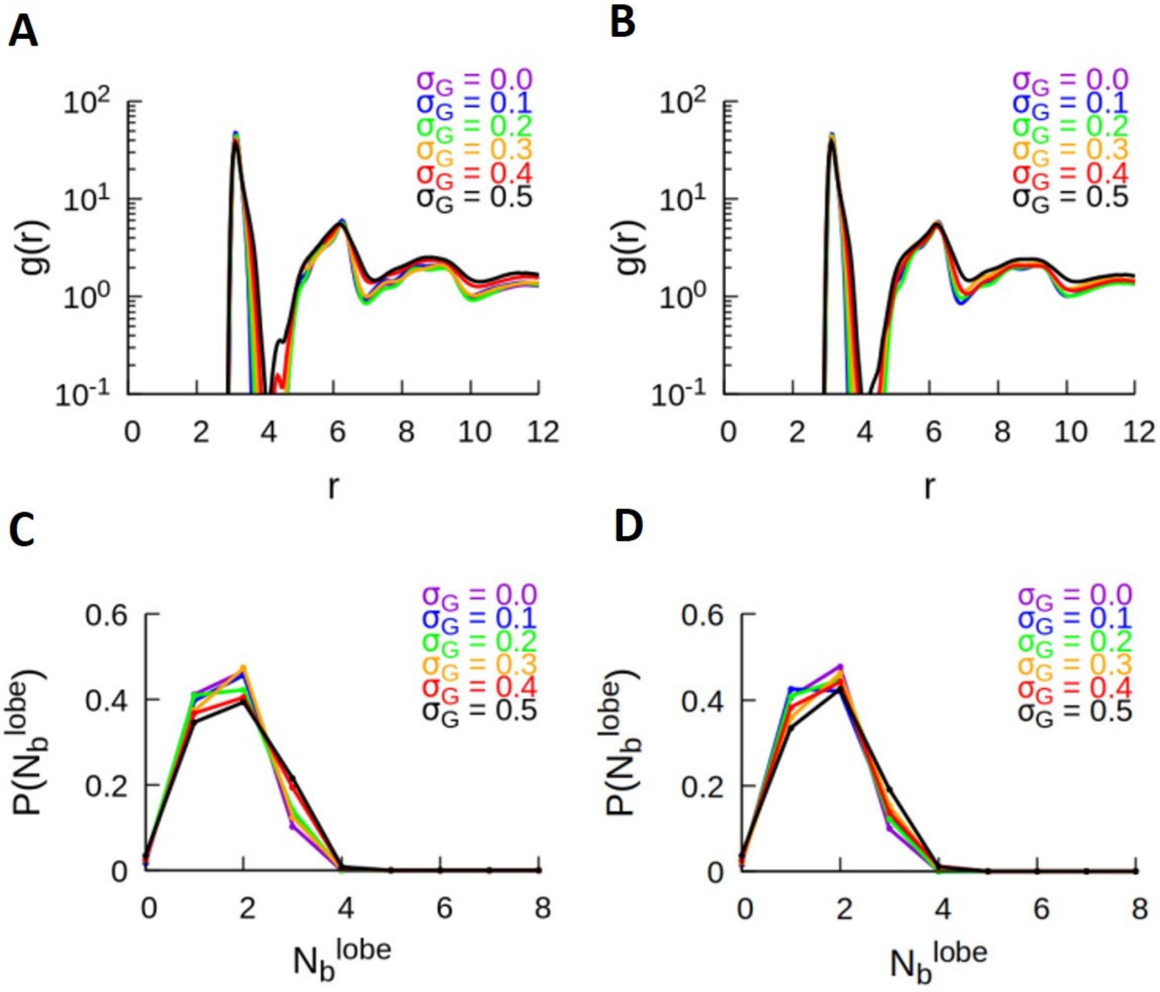

Figure S7: A comparison of results between multiple runs of the DB system at $T^{*} = 0.2$ and $\phi = 0.1$. Shown are the RDF traces for (A) run1, (B) run2, and P(N_b^{lobe}) traces for (C) run1, (D) run2.

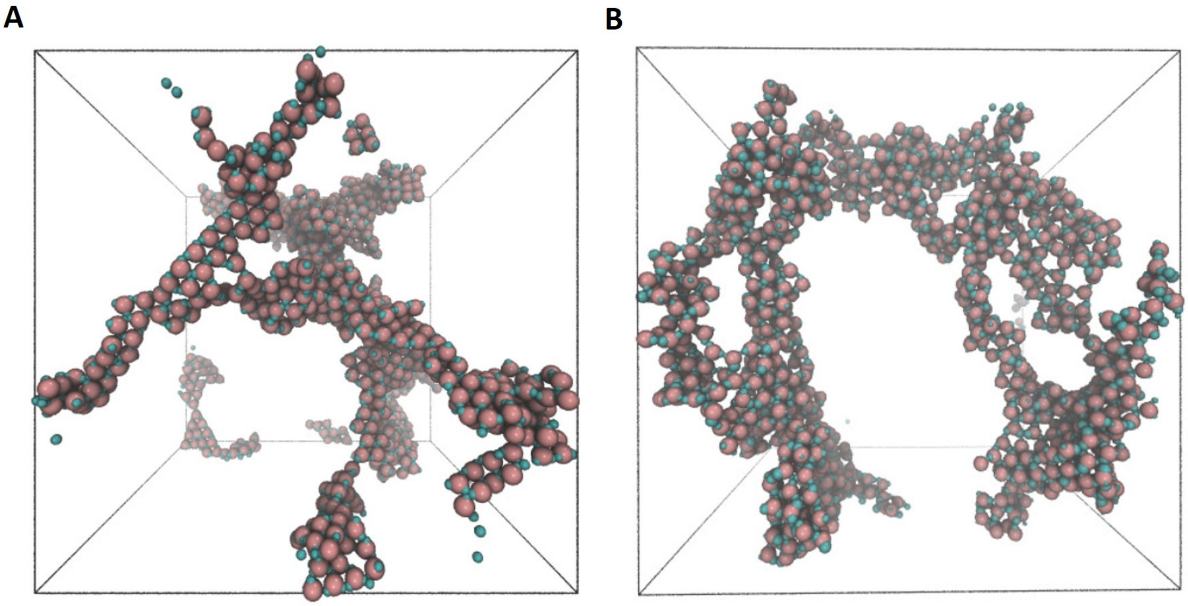

Figure S8: Self-assembly of the TP particles at $T^* = 0.2$. Shown are the snapshots of the interconnected networks observed for different conditions of σ_G: (A) $\sigma_G = 0.1$, and (B) $\sigma_G = 0.5$.

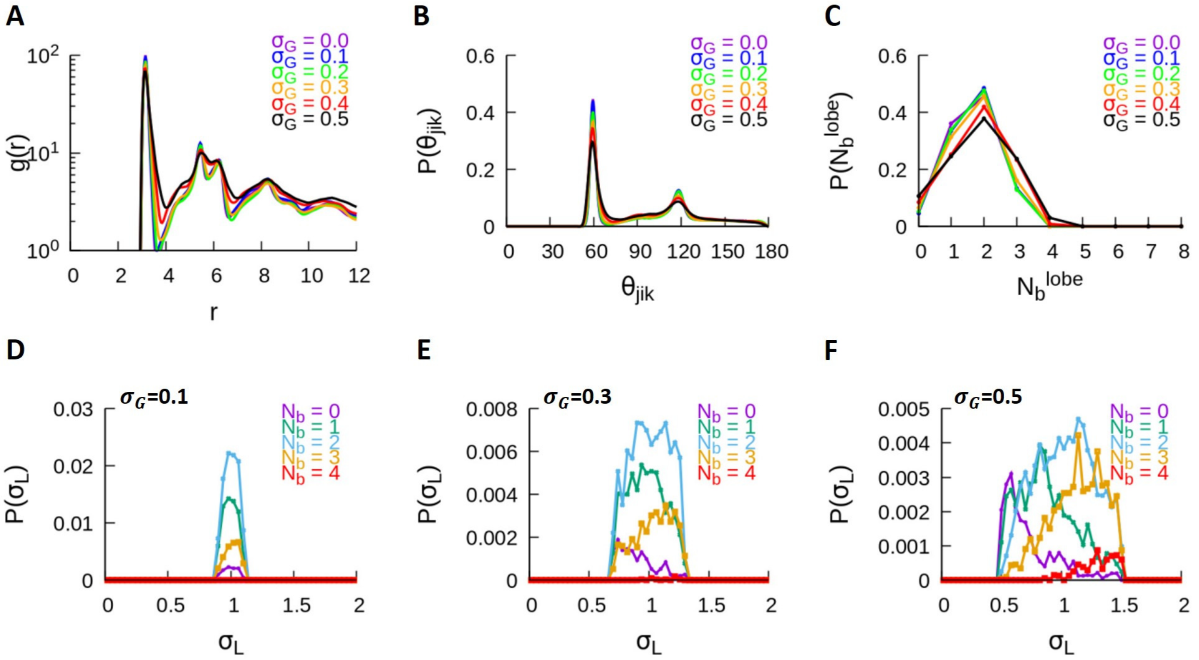

Figure S9: (A-F) Data similar to Figure S3 are shown for the TP system at $T^* = 0.2$.

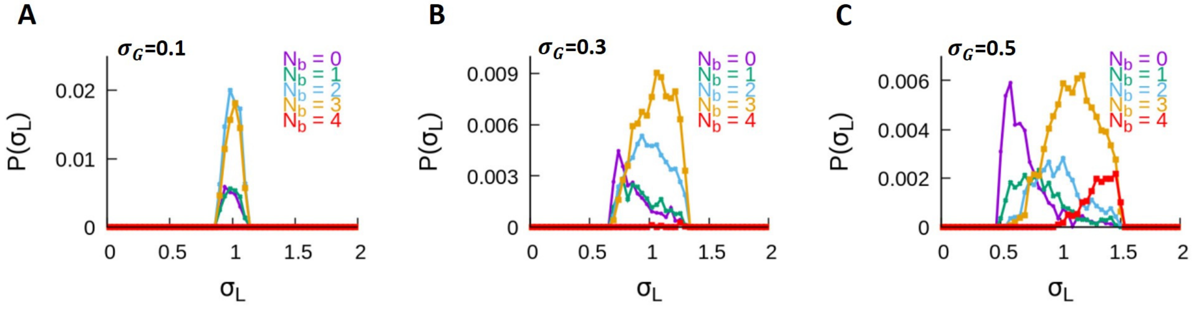

Figure S10: (A-C) Data similar to Figure S2 are shown for the TP system at $T^* = 0.4$.

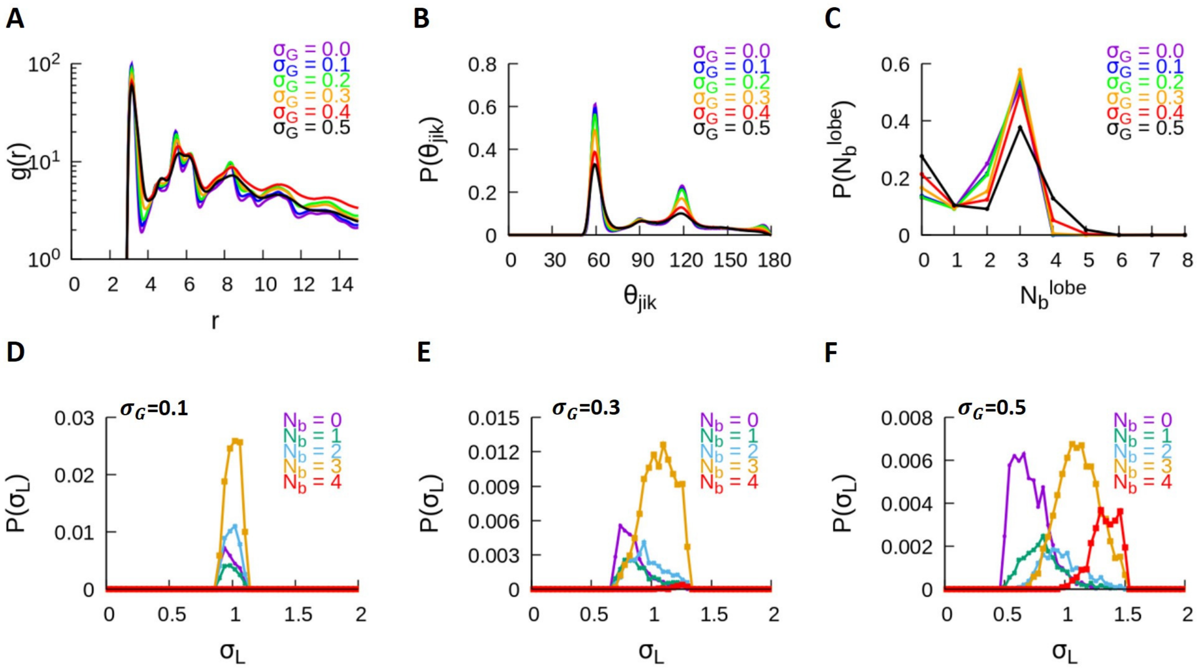

Figure S11: (A-F) Data similar to Figure S3 are shown for the TP system at $T^* = 0.6$.

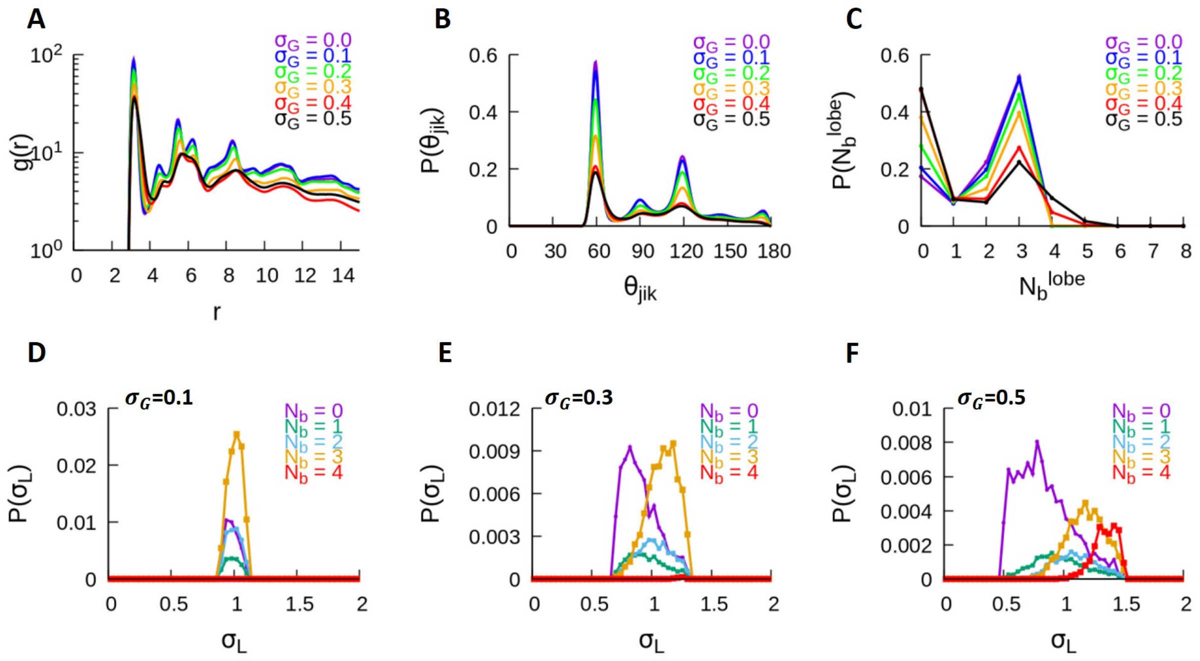

Figure S12: (A-F) Data similar to Figure S3 are shown for the TP system at $T^* = 0.8$.

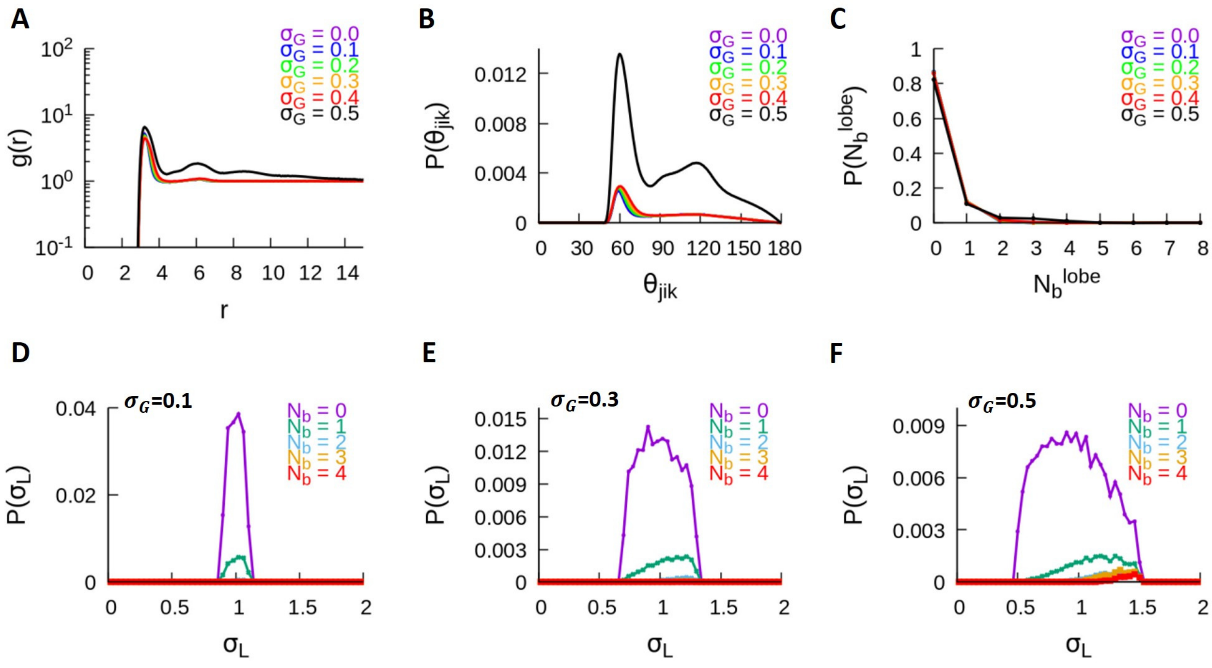

Figure S13: (A-F) Data similar to Figure S3 are shown for the TP system at $T^* = 1.0$.

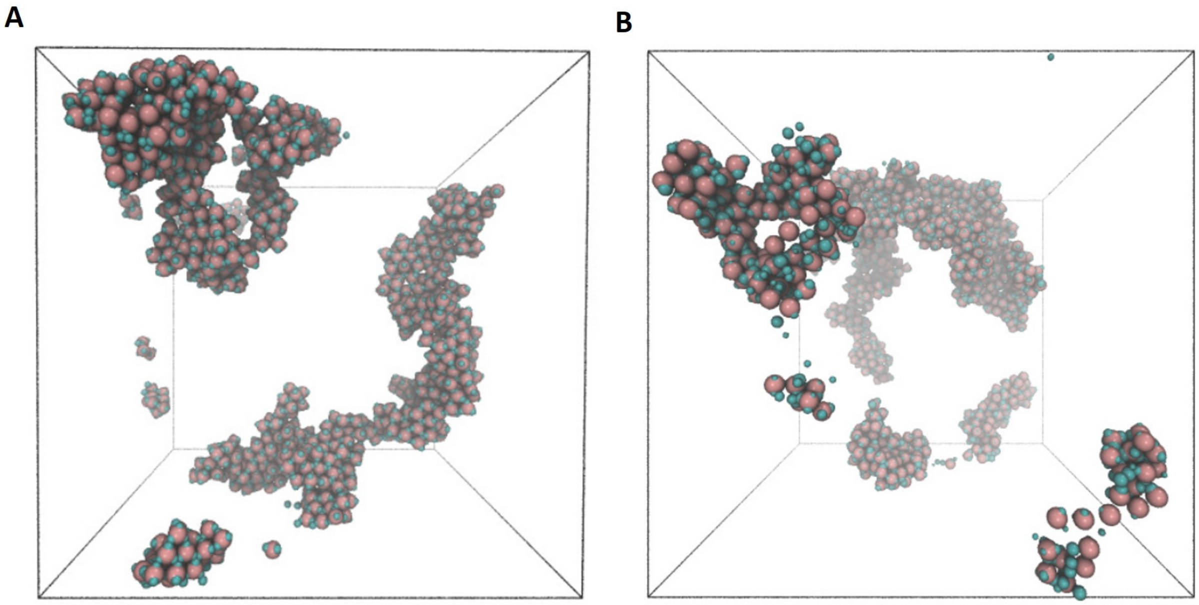

Figure S14: Self-assembly of the TH particles at $T^* = 0.2$. Shown are the snapshots of the interconnected networks observed for different conditions of σ_G: (A) $\sigma_G = 0.1$, and (B) $\sigma_G = 0.5$.

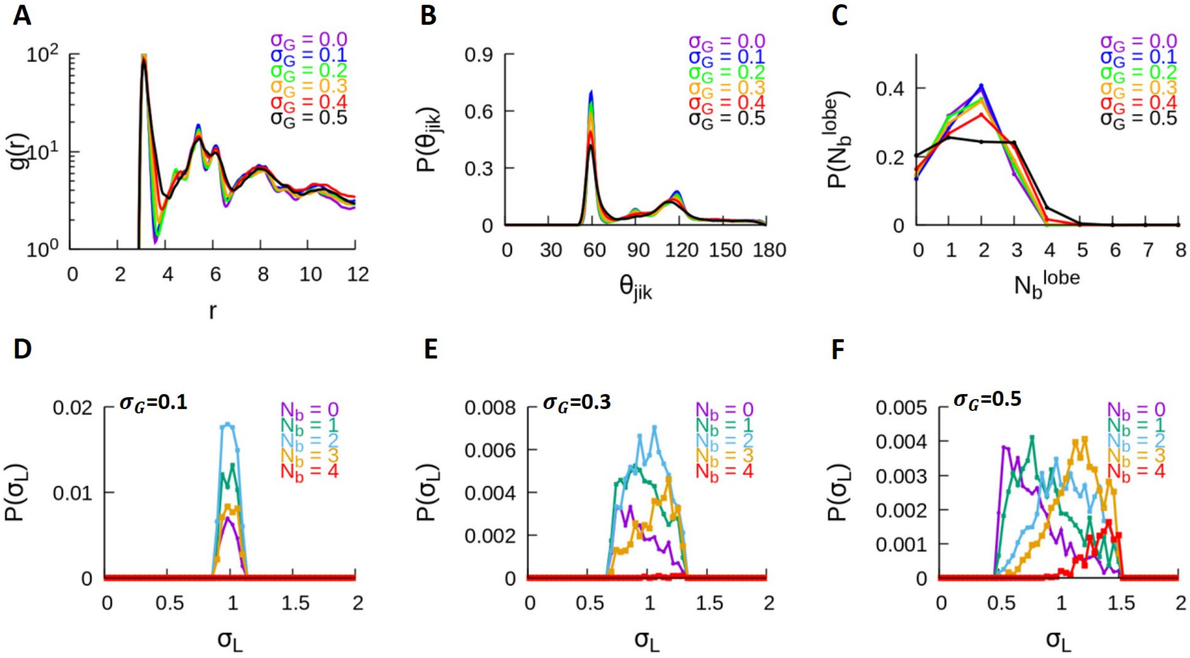

Figure S15: (A-F) Data similar to Figure S3 are shown for the TH system at $T^* = 0.2$.

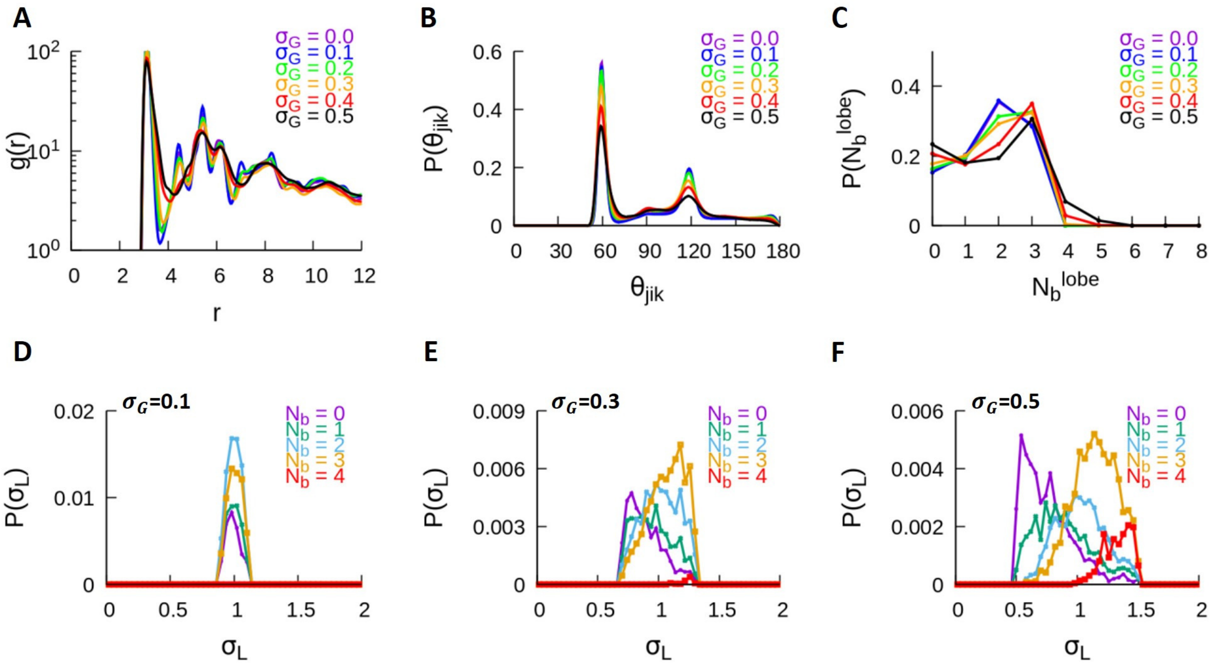

Figure S16: (A-F) Data similar to Figure S3 are shown for the TH system at $T^* = 0.4$.

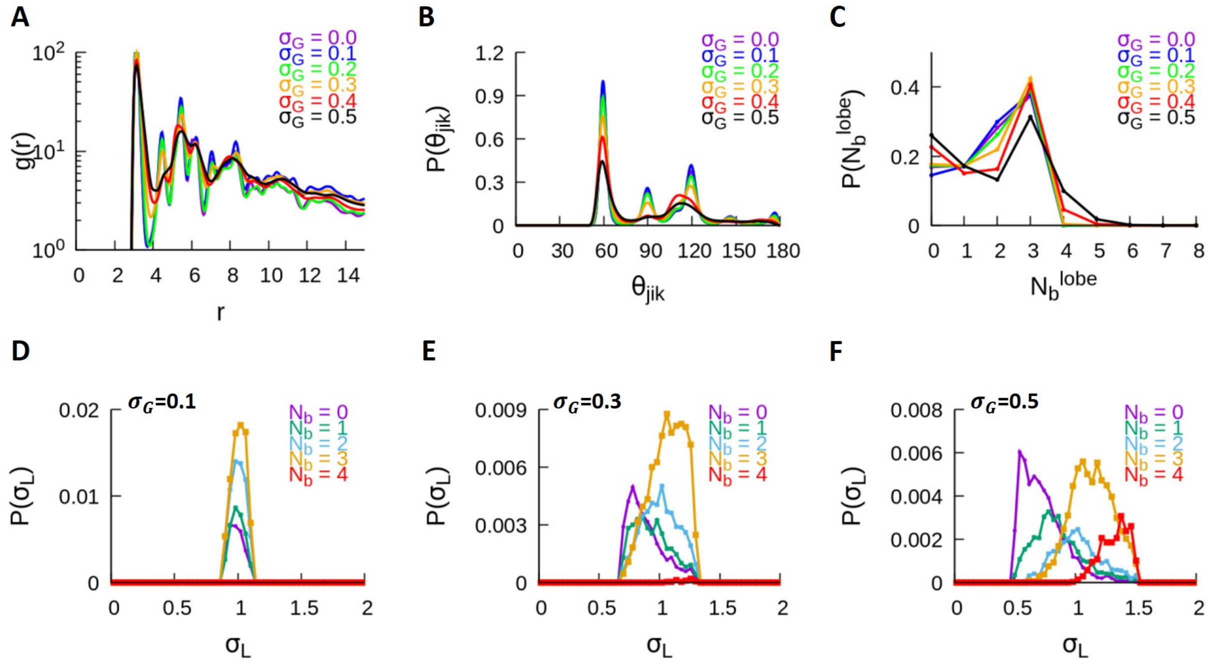

Figure S17: (A-F) Data similar to Figure S3 are shown for the TH system at $T^* = 0.6$.

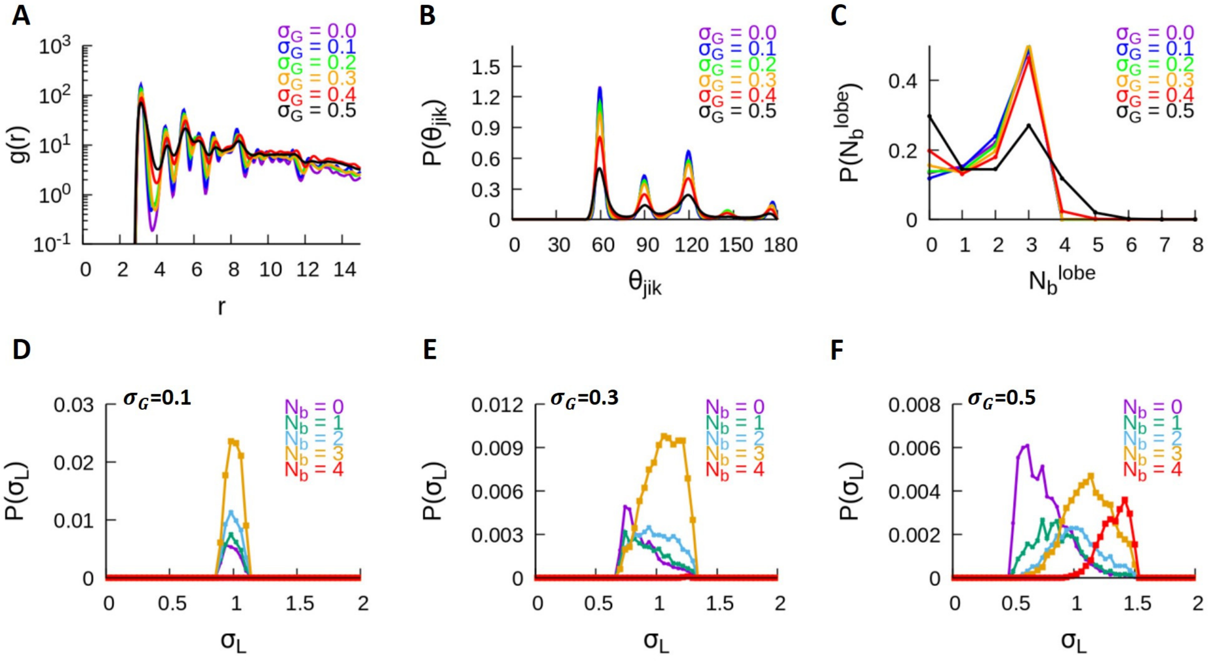

Figure S18: (A-F) Data similar to Figure S3 are shown for the TH system at $T^* = 0.8$.

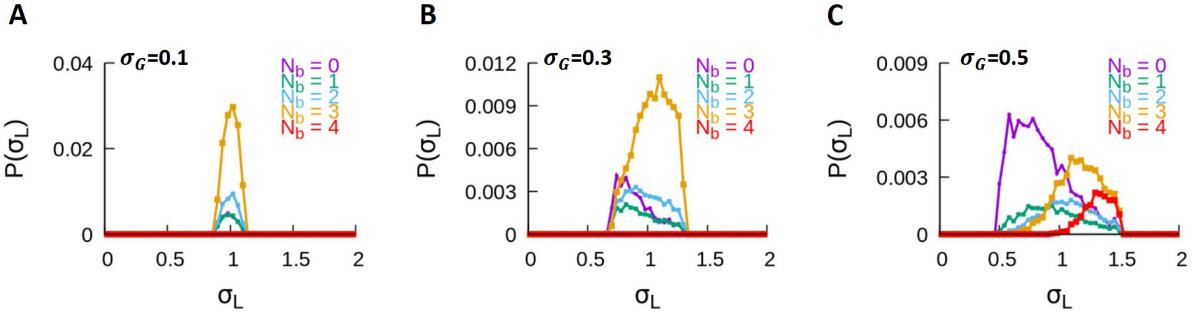

Figure S19: (A-C) Data similar to Figure S2 are shown for the TH system at $T^* = 1.0$.

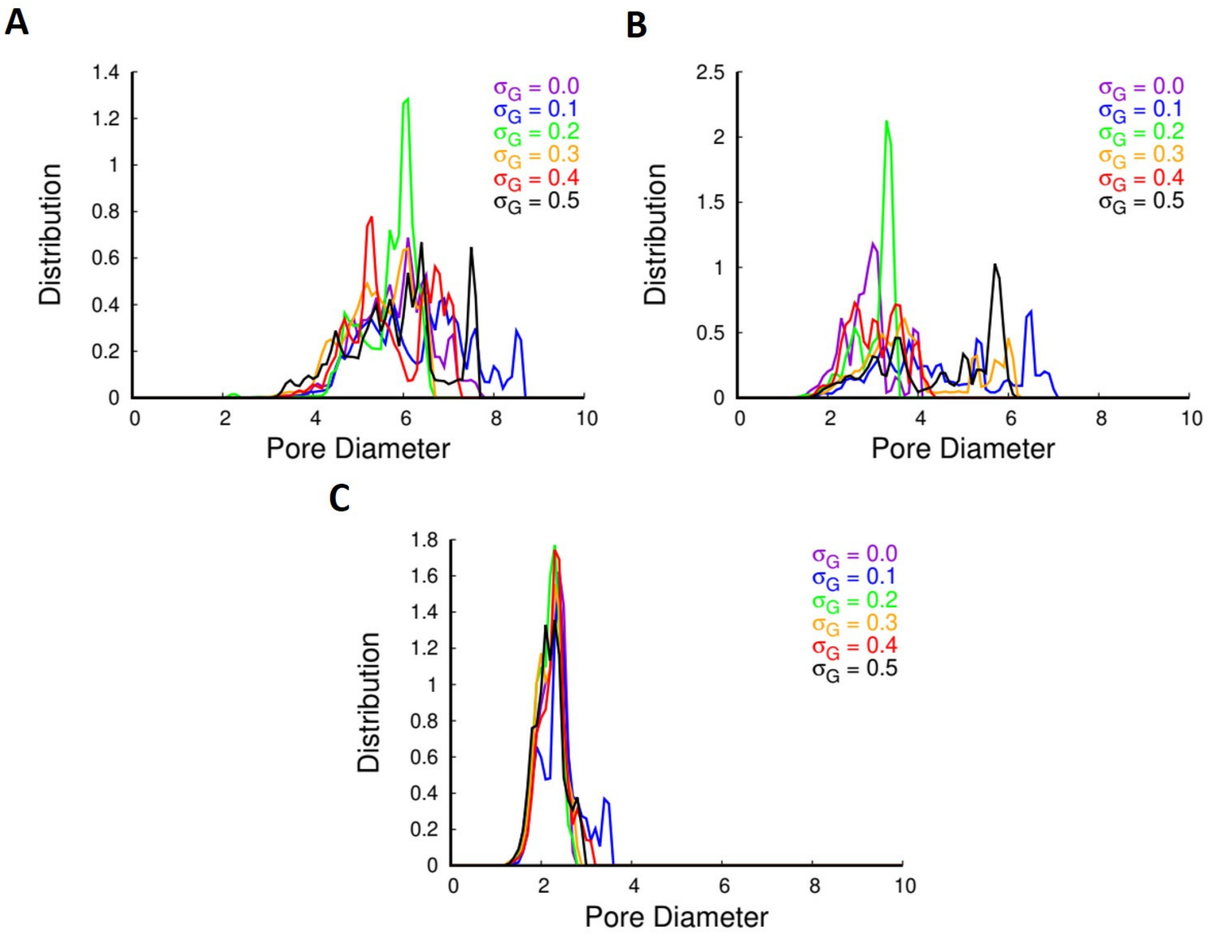

Figure S20: Shown are data on the pore size distributions for three lobed particle systems at different conditions of T^*: (A) DB at T^* = 0.6, (B) TP at T^* = 0.8, and (C) TH at T^* = 1.0.
